# Alterations in Three-dimensional Knee Kinematics and Kinetics during Neutral, Squeeze and Outward Squat

**DOI:** 10.2478/hukin-2013-0068

**Published:** 2013-12-31

**Authors:** Shuyang Han, Shirong Ge, Hongtao Liu, Rong Liu

**Affiliations:** 1 College of Mechanical and Electrical Engineering, China University of Mining and Technology, Xuzhou, China.; 2 College of Materials Science and Engineering, China University of Mining and Technology, Xuzhou, China.

**Keywords:** squat performance, foot angle, knee alignment, rehabilitation, osteoarthritis

## Abstract

The squat exercise was usually performed with varying feet and hip angles by different populations. The objective of this study was to compare and contrast the three-dimensional knee angles, moments, and forces during dynamic squat exercises with varying feet and hip angles. Lower extremity motions and ground reaction forces for fifteen healthy subjects (9 females and 6 males) were recorded while performing the squat with feet pointing straight ahead (neutral squat), 30º feet adduction (squeeze squat) and 30º feet abduction (outward squat). Nonparametric procedures were used to detect differences in the interested measures between the conditions. No significant difference in three-dimensional peak knee angles was observed for three squat exercises (p>0.05), however, the overall tendency of knee rotations was affected by varying feet and hip positions. During the whole cycle, the outward squat mainly displayed adduction moments, while the neutral and squeeze squat demonstrated abduction moments. Peak abduction moments were significantly affected by feet positions (p<0.05). Moreover, the tibiofemoral and patellofemoral joint forces progressively increased as knee flexed and decreased as knee extended, yet peak forces were not affected by varying feet positions (p>0.05). In conclusion, a neutral position is recommended to perform the squat exercise, while the squeeze squat and outward squat might contribute to the occurrence of joint pathologies.

## Introduction

The squat exercise is a classic multiple-joint, closed kinetic chain exercise that plays an important role in improving lower body strength and enhancing performance. It has become an integral part of most musculoskeletal disorders treatment programs (Dionisio et al., 2008; Liu et al., 2010), especially the knee rehabilitation programs (Escamilla, 2001). Meanwhile, as a daily activity, the squat is also widely performed while resting, socializing, toileting and household chores in some non-western cultures (Hemmerich et al., 2006). It was reported that 40% of men and 68% of women in China performed squatting more than 1 hour per day (Zhang et al., 2004). Several studies have investigated the patterns of kinematics (Zeller et al., 2003; Wallace et al., 2008), kinetics (Dionisio et al., 2008; Nagura et al., 2002) and muscle activities (Isear et al., 1997) of the knee joint during the squat for both healthy and pathological subjects (Salem et al., 2003; Liu et al., 2010; Yamazaki et al., 2010). Understanding of the knee biomechanics during the squat would be beneficial for those who are interested in closed kinetic chain exercises, joint rehabilitation and sport training.

It is worth noting that, due to the differences in lifestyle, physical characteristics or requirements of sports activities, the squat was usually performed with various methods by different populations. For instance, females always demonstrated hip adduction and knee valgus during the squat compared with males (Zeller et al., 2003; Yamazaki et al., 2010), which was described as a ‘squeeze squat’ (Earl et al., 2001). Conversely, weightlifters often place their hip and feet into an abduction position while performing a squat snatch (Werner, 1983). This position could also be demonstrated for Asians while resting or toileting (Hemmerich et al., 2006). The valgus and varus alignment would alter load distributions at the knee, thereby contributing to the occurrence of joint pathologies (e.g. knee osteoarthritis (OA) (Brouwer et al., 2007; Kujala et al., 1995), anterior cruciate ligament (ACL) injury (Zeller et al., 2003)). Furthermore, previous studies have shown that the quadriceps, hamstrings, or gastrocnemius activities were independent of feet positions during the squat (Hung and Gross, 1999; Escamilla et al., 2001; Murray et al., 2013).

To our knowledge, only few studies have systematically compared the kinematics and kinetics at the knee during the squat with varying hip and feet positions. A kinematic and kinetic description for the squat will not only improve our understanding of the underlying biomechanics during the squat, but guide the optimization of this task in different sports training and joint rehabilitation programs as well. Therefore, the objective of this study was to synthetically compare and contrast the knee angles, moments, and forces during dynamic squat exercises with varying feet and hip positions. We hypothesized that the changes in feet angles would produce no difference in knee joint kinematics and kinetics.

## Material and Methods

### Participants

A total of 15 healthy volunteers (9 females and 6 males) were recruited for this study from the university students. Their mean age, body height, and body mass were 21.4 ± 2.0 years, 170.4 ± 9.6 cm, and 64.7 ± 11.8 kg, respectively. All subjects had no history of injury or pain in the lower limbs, or balance problems that would affect their performances. This study was approved by the ethics committee of China University of Mining and Technology, and all participants signed a consent form before its commencement.

### Procedures

The Optotrak^®^ Certus™ 3020 dynamic tracking system (Northern Digital Inc., Waterloo, Canada) was employed to capture the kinematic data at a frequency of 100 Hz. Two force plates (Bertec, USA) were used to record the ground reaction forces (GRF). Seven optical tracking rigid plates with each consisting of four markers and one shell were designed according to the body characters and were attached to the lateral aspects of the feet (bilateral instep), shanks (bilateral surface of tibia), thighs (bilateral surface of the thigh), and pelvis (over the center point between both posterior superior iliac spines), respectively.

With each foot on one force plate, the subjects performed the squat exercises under three conditions: (1) both feet pointing straight ahead (neutral squat), (2) hip adduction and 30º of feet adduction (squeeze squat), and (3) hip abduction and 30º of feet abduction (outward squat). The subjects were required to perform each activity from an initial upright position with the feet shoulders width apart, the arms in 90º of shoulder flexion and elbows extension. They were also instructed to maintain the feet in the initial position during the exercise. At a low descending speed, the subjects squatted down until the thighs were parallel with the ground and then in a continuous motion ascended back to the upright position. For each subject, six successful trials were recorded.

Local coordinate systems were defined for the foot, shank, thigh and pelvis segments through digitized palpated bony landmarks. The bony landmarks included left/right ilium anterior superior, left/right prominence of the greater trochanter external surface, left/right femur lateral/medial epicondyle, left/right fibula apex of lateral malleolus, left/right tibia apex of medial malleolus, left/right dorsal aspect of first metatarsal head, and left/right dorsal aspect of fifth metatarsal head. These local coordinate systems enabled the calculation of the floating axis angles at the knee joint (Grood and Suntay, 1983). The raw kinematic data were smoothed using a fourth-order zero lag digital Butterworth low pass filter with cut-off frequency at 6 Hz. Three-dimensional (3D) joint angles, moments and forces were calculated in the Visual 3D software (C-Motion Inc., Rockville, MD, USA) based on the subjects’ lower limbs length, body mass and ground reaction force. The forces and moments were normalized to body weight (BW) and percent of body weight times height (% BW×Ht), respectively.

### Statistical Analysis

To generate ensemble graphs, data throughout a squat cycle were normalized to 101 points (0%–100%). The average measures of every subject were obtained from six trials, and then these individual data were averaged for all subjects. Nonparametric Wilcoxon sign-rank tests were performed using SPSS. Statistical significance was set at *p*<0.05.

## Results

**Figure 1:**
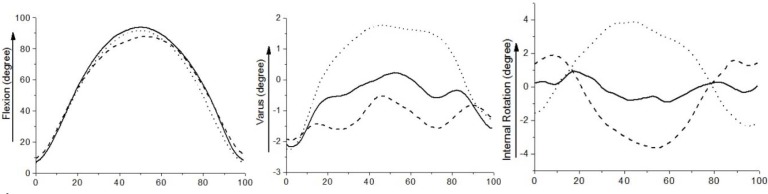
Mean curves of 3D joint angles for three different squat exercises (Solid: neutral squat, Dash: squeeze squat, Dot: outward squat)

Compared to the neutral squat, the outward squat demonstrated an offset towards varus at the knee, whereas the squeeze squat displayed a valgus offset during approximately 10–90% of the squat cycle (Figure 1). Nevertheless, there was no significant difference in 3D peak angles between the two conditions (*p*>0.05) (Table 1). Furthermore, feet and hip abduction induced external rotation of the knee at the upright position and vice versa. The overall tendency of knee rotations was also affected by varying feet positions. With increasing knee flexion, the knee gradually moved towards internal rotation during outward squat. In contrast, the knee was progressively external rotated with increasing knee flexion during the squeeze squat. At approximately 50–60º of the knee flexion, three mean curves of rotational angles crossed each other (Figure 1).

**Figure 2:**
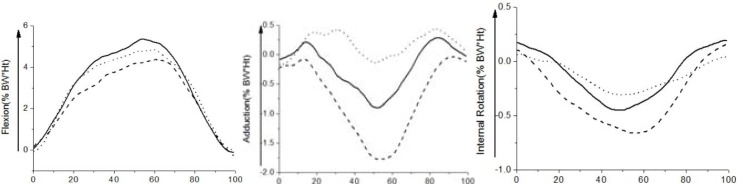
Mean curves of 3D joint moments for three different squat exercises (Solid: neutral squat, Dash: squeeze squat, Dot: outward squat)

**Figure 3:**
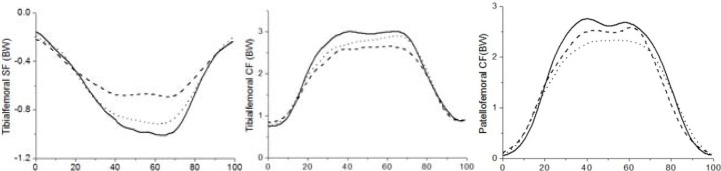
Mean curves of tibiofemoral shear force, compressive force and patellofemoral compressive force for three different squat exercises (Solid: neutral squat, Dash: squeeze squat, Dot: outward squat)

In term of joint moments, the flexion moment gradually increased as the knee flexed and decreased as the knee extended (Figure 2). The differences between the three conditions were mainly distributed between 20–80% of the squat cycle, yet the peak flexion moments were not significantly different between any of the two conditions (*p*>0.05) (Table 2). Peak abduction moments were significantly influenced by feet positions (*p*=0.04 for the neutral squat vs. squeeze squat, *p*=0.03 for the squeeze squat vs. outward squat, and *p*=0.03 for the neutral squat vs. outward squat). Peak adduction moment for the squeeze squat was significantly lower than that for the outward squat (*p*=0.03) (Table 2). Moreover, significant differences were also observed in peak external rotation moments between the outward squat and neutral squat (*p*=0.04), and between the outward squat and squeeze squat (*p*=0.02) (Table 2). On the other hand, both the tibiofemoral shear force (SF) and the compressive force (CF) progressively increased in the descent phase and decreased in the ascent phase (Figure 3). Statistical analysis indicated that no difference was found in the tibiofemoral forces for the neutral, squeeze and outward squat (Table 3). Similarly, the patellofemoral forces increased in the descent phase and decreased in the ascent phase, being maximal at peak knee flexion. The differences in peak patellofemoral forces were not statistically significant among the three kinds of squats (Figure 3).

## Discussion

The objective of this study was to compare and contrast the knee kinematics and kinetics during the neutral squat, squeeze squat and outward squat, thereby improving our understanding of knee biomechanics during the squat, and providing beneficial information for sports training and joint rehabilitation programs.

Because of varied feet and hip positions, there were valgus/varus alignments at the knee. The squeeze squat demonstrated a valgus knee, while the outward squat displayed a varus knee. Changes in rotation angles were also observed for the squeeze and outward squat at the initial upright position. 30º feet abduction demonstrated an internally rotated knee and 30º feet adduction externally rotated the knee. Moreover, it had been reported that the screw home mechanism changed during the squat exercise, both the femur and the tibia tended to rotate externally during the descent phase and internally during the ascent phase (Escamilla, 2001). This was true for the neutral and squeeze squat in this study. However, for the outward squat, the knee joint was internally rotated as the knee flexed and externally rotated as the knee extended. Similar results were demonstrated during walking. With valgus alignment, the knee demonstrated an offset towards external rotation during the swing phase, whereas the knee was internally rotated with varus alignment (Werner et al., 2005). During the squeeze and outward squat, the extreme external or internal rotation at maximal knee flexion would cause the anterior or posterior portions of the menisci to be compressed. This could produce a twisting strain on the collateral ligaments, and be injurious to the meniscus (Escamilla, 2001). Therefore, the neutral squat is more healthy and safe for the knee, while the repetitive squeeze and outward squat might potentially cause meniscus injury.

The knee adduction moment has been identified as an important factor in the development and progression of OA. In this study, significant differences in peak adduction/abduction moments were demonstrated with knee alignments for three squat exercises. This is consistent with the results during walking (Wada et al., 2001; Chang et al., 2004). Compared to the neutral squat, elevated abduction moment was observed through most of the squat cycle for the squeeze squat, while hardly any abduction moment was observed throughout the outward squat. The greater abduction moment during the squeeze squat would produce more loads on the lateral compartment, while the adduction moment generates more medial compartment loads during the outward squat (Baliunas, 2002). The load changes could induce a high risk of joint OA, as it has been indicated that an increase of 1% in knee adduction moment would raise the risk of progression by 6.46 times (Foroughi et al., 2009). Therefore, the neutral squat is a preferred performance for joint rehabilitation or sports training, while the squeeze squat and outward squat should be avoided.

Tibiofemoral SF and CF play an important role in cruciate ligaments injury, meniscal tear and articular cartilage degeneration. In the present study, the tibiofemoral SF and CF increased as the knee flexed and decreased as the knee extended, with peak forces occurring at maximal knee flexion, being consistent with several other studies (Escamilla et al., 2001; Stuart et al., 1996; Wilk et al., 1996). The results of this study indicated that both tibiofemoral SF and CF were not significantly affected by varying feet and hip angles, which was in agreement with the findings for the barbell squat (Escamilla et al., 2001). Besides, similarly to several studies (Stuart et al., 1996; Wilk et al., 1996; Escamilla et al., 2001), the tibiofemoral SF was consistently in a posterior direction throughout the squat cycle, while no anterior SF was observed. In some other studies, only small anterior SF was reported (Toutoungi et al., 2000). The low or absence of anterior SF was probably due to the combined effects of hamstring and quadriceps (Escamilla, 2001). Because of generating small anterior SF, the squat exercise could be an effective exercise for those who need to minimize tensile loading of the ACL. In this study, the peak tibiofemoral CF ranged from 2.99 times BW to 3.32 times BW, which was in agreement with previous results (Escamilla, 2001). For the squeeze and outward squat, there was a tendency of lower tibiofemoral CF compared with the neutral squat, although the differences did not achieve the level of significance. The tibiofemoral CF was considered to be a key factor in the development and progression of OA. It was reported that the prevalence of knee OA was equal to 30% among soccer players and weightlifters, who always bear excessive tibiofemoral loads (Kujala et al., 1995). Within the range of 50–60º of knee flexion, the tibiofemoral CF was not more than 1.5 times BW for all three squat exercises, but it showed a nearly linear increase from 60º to maximal knee flexion. Thus, individuals who suffer from tibiofemoral pathologies should avoid performing the squat at high flexion angles.

It has been reported that the CF was slightly higher during the descent phase compared with the ascent phase (Escamilla et al., 1998). The same result was demonstrated for the neutral squat, but not for the squeeze squat and outward squat in this study. With increasing knee flexion, both the patellar tendon and quadriceps tendon contribute more to the forces in the compressive direction, thus the patellofemoral CF increases steadily as the knee flexed. However, the patellofemoral contact remains fairly constant from 60 to 90º of knee flexion (Wallace et al., 2002). As a consequence, joint stress, which is defined as the CF divided by contact area, could be injurious to the articular contact surface. Therefore, the squat exercise should not be performed at high flexion angles for those who have patellofemoral joint pathologies. Besides, repetitive occurrences of a high flexion squat and a prolonged squat could also be strong risk factors for tibiofemoral and patellofemoral joint (Zhang et al., 2004; Kujala et al., 1995).

Several limitations should be pointed out in the present study. First, muscle activities were not evaluated in this paper, although several other studies have shown that the muscle activity was independent of the feet angles during the squat (Hung and Gross, 1999; Escamilla et al., 2001; Murray et al., 2013). Second, compared to the neutral squat, the squeeze squat and outward squat were less performed by the subjects in their daily life. This unfamiliarity might bring errors into the results. Third, females have been reported having a more valgus knee compared to males because of a wider pelvis (Zeller et al., 2003). The differences in physical characteristics might result in different performances for the males and females. However, the influence of gender on the results was not taken into consideration in this study.

In conclusion, varying feet and hip angles affected the knee joint rotations and adduction moments during the squat, while the tibiofemoral and patellofemoral forces were similar among three squat exercises. The neutral squat is a preferred method for joint rehabilitation and sports training, while the squeeze squat and outward squat might contribute to the occurrence of joint pathologies. Meanwhile, the dynamic squat exercise is an effective rehabilitation program for patients after an ACL injury or reconstruction, and should be performed at low knee flexion by those who suffered from tibiofemoral and patellofemoral pathologies.

## Practical implications for sports training

The squat exercise could be performed preferably in a neutral position, especially when it is performed with heavy resistance. Compared to the squeeze squat and outward squat, the neutral squat could help prevent the lower limbs from joint pathologies.

## Figures and Tables

**Table 1:** Mean (±SD) peak joint angles for three squat exercises

**Angles (degree)**	Neutral Squat	Squeeze Squat	Outward Squat
Maximum flexion	94.7±10.58	88.9±12.95	91.9±14.15
Minimum flexion	6.8±5.10	8.99±7.39	4.20±5.21
Maximum valgus	0.3±3.73	−0.4±4.66	1.8±3.89
Maximum varus	2.2±4.17	2.0±4.56	2.2±4.06
Maximum internal rotation	1.1±5.60	1.9±6.06	3.9±5.46
Maximum external rotation	1.0±7.26	3.6±8.38	2.3±3.57

**Table 2:** Mean (±SD) peak joint moments for three squat exercises

**Moments (%BW × Ht)**	Neutral Squat	Squeeze Squat	Outward Squat
Minimum flexion	0.4±0.57	0.5±0.53	0.2±0.36
Maximum flexion	5.7±1.92	4.6±1.85	4.9±2.00
Maximum adduction	0.5±0.59	0.1±0.43^#^	0.9±0.43^#^
Maximum abduction	1.2±0.65	1.9±0.10^*#^	0.6±0.40^*#^
Maximum internal rotation	0.2±0.17	0.2±0.12	0.1±0.19
Maximum external rotation	0.5±0.22	0.7±0.33^#^	0.4±0.31^*#^

* Significant different from neutral squat

# Significant different from squeeze (outward) squat

**Table 3:** Mean (±SD) peak joint forces for three squat exercises

**Peak Forces (BW)**	Neutral Squat	Squeeze Squat	Outward Squat
Tibiofemoral SF (posterior)	0.92±0.29	0.73±0.20	1.04±0.21
Tibiofemoral CF	3.32±0.62	2.99±0.89	3.12±0.74
Patellofemoral CF	3.85±0.81	3.64±1.1	3.19±0.33

## References

[b1-jhk-39-59] Baliunas A (2002). Increased knee joint loads during walking are present in subjects with knee osteoarthritis. Osteoarthr Cartilage.

[b2-jhk-39-59] Brouwer GM, van Tol AW, Bergink AP, Belo JN, Bernsen RM, Reijman M, Pols HA, Bierma-Zeinstra SM (2007). Association between valgus and varus alignment and the development and progression of radiographic osteoarthritis of the knee. Arthritis Rheum.

[b3-jhk-39-59] Chang A, Hayes K, Dunlop D, Hurwitz D, Song J, Cahue S, Genge R, Sharma L (2004). Thrust during ambulation and the progression of knee osteoarthritis. Arthritis Rheum.

[b4-jhk-39-59] Dionisio VC, Almeida GL, Duarte M, Hirata RP (2008). Kinematic, kinetic and EMG patterns during downward squatting. J Electromyogr Kinesiol.

[b5-jhk-39-59] Earl JE, Schmitz RJ, Arnold BL (2001). Activation of the VMO and VL during dynamic mini-squat exercises with and without isometric hip adduction. J Electromyogr Kinesiol.

[b6-jhk-39-59] Escamilla RF (2001). Knee biomechanics of the dynamic squat exercise. Med Sci Sport Exer.

[b7-jhk-39-59] Escamilla RF, Fleisig GS, Zheng N, Barrentine SW, Wilk KE, Andrews JR (1998). Biomechanics of the knee during closed kinetic chain and open kinetic chain exercises. Med Sci Sport Exer.

[b8-jhk-39-59] Escamilla RF, Fleisig GS, Zheng N, Lander JE, Barrentine SW, Andrews JR, Bergemann BW, Moorman CT (2001). Effects of technique variations on knee biomechanics during the squat and leg press. Med Sci Sport Exer.

[b9-jhk-39-59] Foroughi N, Smith R, Vanwanseele B (2009). The association of external knee adduction moment with biomechanical variables in osteoarthritis: a systematic review. The Knee.

[b10-jhk-39-59] Grood ES, Suntay WJ (1983). A joint coordinate system for the clinical description of three-dimensional motions: application to the knee. J Biomech Eng.

[b11-jhk-39-59] Hemmerich A, Brown H, Smith S, Marthandam SSK, Wyss UP (2006). Hip, knee, and ankle kinematics of high range of motion activities of daily living. J Orthopaed Res.

[b12-jhk-39-59] Hung YJ, Gross MT (1999). Effect of foot position on electromyographic activity of the vastus medialis oblique and vastus lateralis during lower-extremity weight-bearing activities. J Orthop Sport Phys.

[b13-jhk-39-59] Isear JA, Erickson JC, Worrell TW (1997). EMG analysis of lower extremity muscle recruitment patterns during an unloaded squat. Med Sci Sport Exer.

[b14-jhk-39-59] Kujala UM, Kettunen J, Paananen H, Aalto T, Battié MC, Impivaara O, Videman T, Sarna S (1995). Knee osteoarthritis in former runners, soccer players, weight lifters, and shooters. Arthritis Rheum.

[b15-jhk-39-59] Liu MF, Chou PH, Liaw LJ, Su FC (2010). Lower-limb adaptation during squatting after isolated posterior cruciate ligament injuries. Clin Biomech.

[b16-jhk-39-59] Murray N, Cipriani D, O'rand D, Reed-Jones R (2013). Effects of foot position during squatting on the quadriceps femoris: an electromyographic study. Int J Exer Sci.

[b17-jhk-39-59] Nagura T, Dyrby OC, Alexander JE, Andriacchi PT (2002). Mechanical loads at the knee joint during deep flexion. J Orthopaed Res.

[b18-jhk-39-59] Salem GJ, Salinas R, Harding FV (2003). Bilateral kinematic and kinetic analysis of the squat exercise after anterior cruciate ligament reconstruction. Arch Phys Med Rehabil.

[b19-jhk-39-59] Stuart MJ, Meglan DA, Lutz GE, Growney ES, An KN (1996). Comparison of intersegmental tibiofemoral joint forces and muscle activity during various closed kinetic chain exercises. Am J Sport Med.

[b20-jhk-39-59] Toutoungi DE, Lu TW, Leardini A, Catani F, O’Connor JJ (2000). Cruciate ligament forces in the human knee during rehabilitation exercises. Clin Biomech.

[b21-jhk-39-59] Wada M, Maezawa Y, Baba H, Shimada S, Sasaki S, Nose Y (2001). Relationships among bone mineral densities, static alignment and dynamic load in patients with medial compartment knee osteoarthritis. Rheumatology.

[b22-jhk-39-59] Wallace BJ, Kernozek TW, Mikat RP, Wright GA, Simons SZ, Wallace KL (2008). A comparison between back squat exercise and vertical jump kinematics: implications for determining anterior cruciate ligament injury risk. J Strength Cond Res.

[b23-jhk-39-59] Wallace DA, Salem GJ, Salinas R, Powers CM (2002). Patellofemoral joint kinetics while squatting with and without an external load. J Orthop Sport Phys.

[b24-jhk-39-59] Werner FW, Ayers DC, Maletsky LP, Rullkoetter PJ (2005). The effect of valgus/varus malalignment on load distribution in total knee replacements. J Biomech.

[b25-jhk-39-59] Werner M (1983). The Knee: Form, Function, and Ligament Reconstruction.

[b26-jhk-39-59] Wilk KE, Escamilla RF, Fleisig GS, Barrentine SW, Andrews JR, Boyd ML (1996). A comparison of tibiofemoral joint forces and electromyographic activity during open and closed kinetic chain exercises. Am J Sport Med.

[b27-jhk-39-59] Yamazaki J, Muneta T, Ju YJ, Sekiya I (2010). Differences in kinematics of single leg squatting between anterior cruciate ligament-injured patients and healthy controls. Knee Surg Sport Tr A.

[b28-jhk-39-59] Zeller BL, McCrory JL, Kibler WB, Uhl TL (2003). Differences in kinematics and electromyographic activity between men and women during the single-legged squat. Am J Sport Med.

[b29-jhk-39-59] Zhang Y, Hunter DJ, Nevitt MC, Xu L, Niu J, Lui LY, Yu W, Aliabadi P, Felson DT (2004). Association of squatting with increased prevalence of radiographic tibiofemoral knee osteoarthritis: the Beijing Osteoarthritis Study. Arthritis Rheum.

